# P-975. Pharmacist Led Vancomycin Dose Optimization at a Tertiary Care Hospital in Addis Ababa, Ethiopia

**DOI:** 10.1093/ofid/ofaf695.1174

**Published:** 2026-01-11

**Authors:** Mahlet Moges, Pineal Yitbarek, Brent W Footer, Katherine Morgan, Benyam Muluneh

**Affiliations:** Addis Ababa University College of Health Sciences, Addis Ababa, Adis Abeba, Ethiopia; Addis Ababa University College of Health Sciences, Addis Ababa, Adis Abeba, Ethiopia; University of North Carolina Medical Center, Chapel Hill, North Carolina; University of North Carolina Medical Center, Chapel Hill, North Carolina; University of North Carolina Eshelman School of Pharmacy, Chapel Hill, North Carolina

## Abstract

**Background:**

Patient-specific vancomycin dosing is paramount to achieving clinical success and reducing toxicity. While pharmacist-guided dosing is utilized in many parts of the world, evidence supporting this practice in African populations is lacking. This study is one of the first interventional studies to assess the impact of pharmacist led Vancomycin dosing among Black communities throughout Africa.

**Figure 1.**
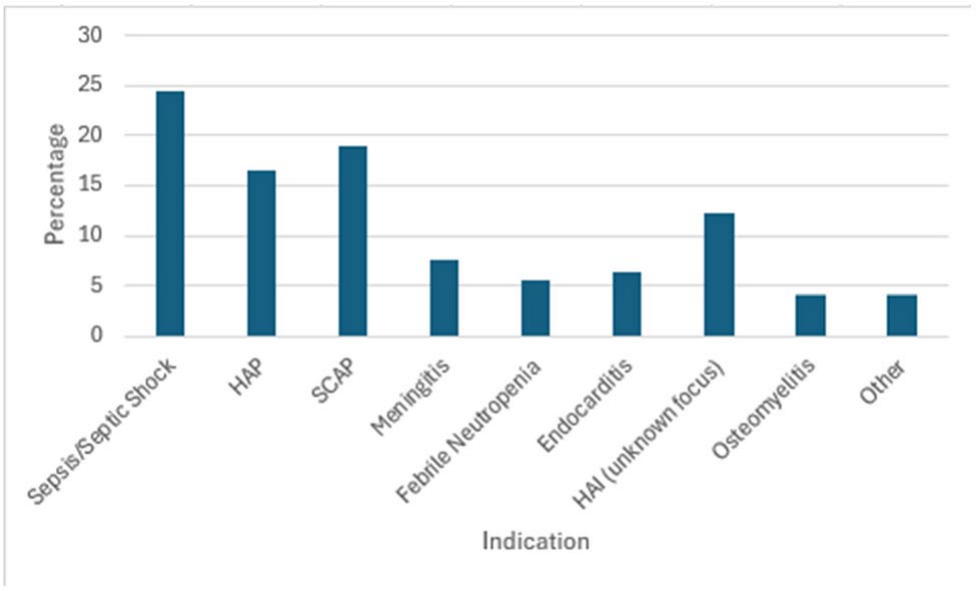
Indications for Vancomycin Therapy HAP = hospital acquired pneumonia, SCAP = severe community acquired pneumonia, HAI = healthcare associated infection

**Table 1.**
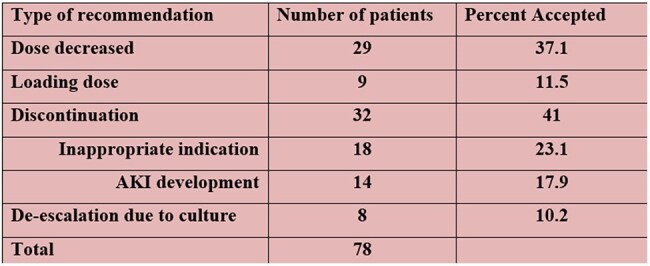
Accepted Clinical Pharmacist Interventions AKI = acute kidney injury

**Methods:**

This prospective interventional study was conducted at Black Lion Hospital, a tertiary care teaching hospital in Addis Ababa, Ethiopia. Pharmacists implemented a vancomycin nomogram for patients admitted to the internal medicine and emergency wards from June 2024 to April 2025. Patients were followed for the entire vancomycin course with adjustments made as necessary.

Pre-implementation most patients received vancomycin at a fixed dose of 1g every 12 hours. Patients with chronic kidney disease or acute kidney injury (AKI), received 1g every 72hr. Post- implementation clinical pharmacists recommended loading and maintenance doses as well as assessed indications, duration of therapy, de-escalation opportunities, and concomitant nephrotoxic medications.

The percentage of physician acceptance of pharmacists’ recommendations was recorded as part of the evaluation.

**Figure 2.**
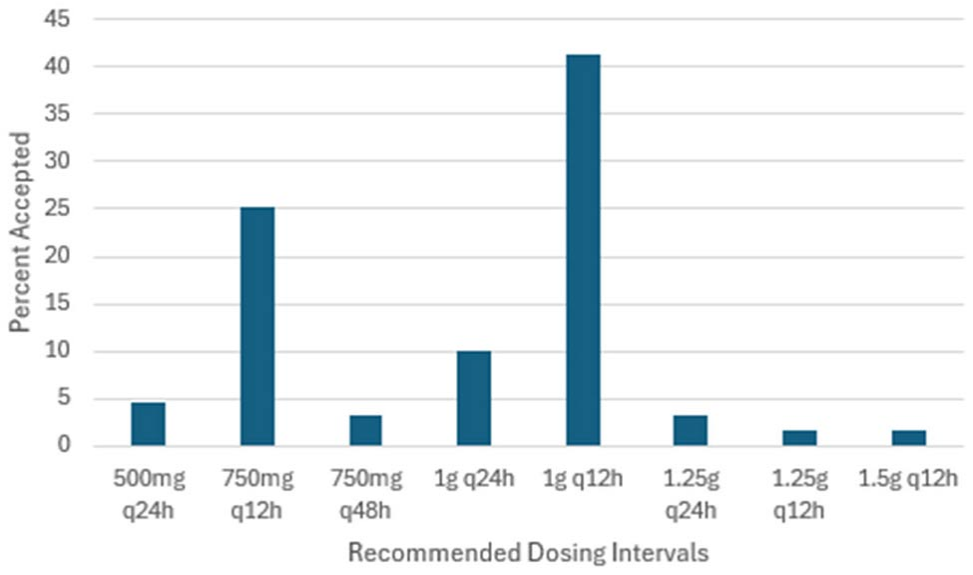
Percentage of Accepted Nomogram Dosing

**Results:**

A total of 144 patients received vancomycin with sepsis/septic shock the most common indication (Figure 1). The mean duration of therapy was 10.7 days. Concomitant nephrotoxic drugs were used in 60.5% of the patients.

Clinical pharmacists made 129 recommendations, of which 78 (51.2%) were accepted. The most common interventions were discontinuation (41%) and decrease of maintenance dose (37.1%) (Table 1). The most accepted dosing regimens were 750mg twice daily (25.3%) and 1g once daily (10%) (Figure3). Recommendations for de-escalation and loading doses were accepted in 10.2% and 11.5% of cases, respectively.

**Conclusion:**

Our findings have significant ramifications for clinical practice, especially in African healthcare settings where established dosing procedures are frequently lacking and empirical vancomycin use is widespread. These findings support wider implementation of pharmacist-driven dosing and optimization strategies.

**Disclosures:**

All Authors: No reported disclosures

